# Delivery outcomes in women with previous caesarean delivery at Zithulele Hospital – A rural district hospital managed by generalist doctors

**DOI:** 10.4102/safp.v68i1.6309

**Published:** 2026-04-22

**Authors:** John D. Michell, Parimalaranie Yogeswaran, Zisiwe Mahlati

**Affiliations:** 1 Department of Family Medicine, Faculty of Medicine and Health Sciences, Walter Sisulu University, Mthatha, South Africa; 2 Department of Public Health, Faculty of Medicine and Health Sciences, Walter Sisulu University, Mthatha, South Africa

**Keywords:** caesarean delivery, vaginal birth after caesarean, trial of labour after caesarean, maternal morbidity, neonatal morbidity

## Abstract

**Background:**

Women with previous caesarean deliveries (CD) contribute to rising CD rates globally, yet limited evidence exists from rural South African district hospitals. The social value of this study lay in understanding delivery outcomes and safety of vaginal birth after caesarean (VBAC) in a resource-limited, generalist clinician setting. The study addressed a knowledge gap regarding VBAC practices and feasibility outside tertiary centres in rural South Africa.

**Methods:**

A descriptive study using a retrospective chart review was conducted at Zithulele Hospital, Eastern Cape. Women with previous CD who delivered at the hospital were included. Data were extracted using a data collection form and analysed using descriptive statistics and comparative tests to identify trends and outcomes in the cohort.

**Results:**

A total of 195 records were analysed. The overall hospital CD rate was 23.1%, with women with scarred uteri (Robson group 5) accounting for 25% of all CD. Forty-six per cent achieved VBAC, while 54% delivered by repeat CD; all women with two or more previous CD delivered by CD. Successful VBAC was associated with prior VBAC (*p* = 0.001) and advanced cervical dilatation at presentation (*p* < 0.001). Maternal complications occurred in 13% of studied population, with 69% following CD.

**Conclusion:**

Vaginal birth after caesarean was feasible, safe and moderately successful in this rural setting. Caesarean delivery contributed disproportionately to overall CD rates, largely because of scar-related indications.

**Contribution:**

This study provides evidence from a rural clinical context supporting VBAC in district hospitals, highlighting practical factors that strengthen counselling and clinical decision-making within district-level obstetric practice.

## Introduction

Caesarean delivery (CD) rates have increased worldwide over the past decades, with sub-Saharan Africa no exception in this trend.^1^ While the World Health Organization (WHO) maintains that evidence is lacking for mortality benefit – whether maternal or perinatal – for a CD rate of more than 10% – 15%, it emphasises that a more reasonable goal would be clinically well-indicated CDs.^2^ The combined South African public and private sector CD rates are now in excess of 28%, according to recent Saving Mothers reports.^3^

While CD can be life-saving, unnecessary procedures expose women to avoidable morbidity, including infection, haemorrhage, adhesions and long-term risks such as abnormal placentation and uterine rupture in subsequent pregnancies, and death.^4^ This effect is more pronounced in low-resource hospitals.^5,6,7^

Safe promotion of vaginal birth after caesarean (VBAC) could reduce the burden of repeat surgery with its inherent high risk of complications thus improving overall maternal and neonatal care. Failed trial of labour after caesarean (TOLAC) has been shown to carry a slightly higher morbidity than the alternative – elective repeat CD – although the absolute risk remains low. Uterine rupture and other birth-related complications are possible complications and skills to handle such should always be accessible.^8,9,10^

For rural district hospital, Maternity units such as at Zithulele Hospital, operating with limited resources and staffed by generalist doctors and midwives, understanding and managing these patterns and risks – both short term and long term – carries important public health relevance. International evidence from tertiary hospitals supports VBAC as a safe and cost-effective alternative with careful selection of patients,^8,9,10,11^ but there remains a paucity of data from rural, generalist-run hospital.

Available South African research on VBAC outcomes originate primarily from tertiary and regional referral hospitals, with specialist-obstetricians oversight.^12,13^ However, there is limited understanding of VBAC feasibility, safety and determinants of success in smaller, district hospitals. The operational environment in district hospitals differs substantially from that of referral centres, particularly with regard to staffing profiles – often characterised by more junior clinicians and generalist practitioners – as well as longer transfer times to higher levels of care when complications arise. These contextual differences may influence both clinical decision-making and maternal and neonatal outcomes following a TOLAC.

This highlights the need for context-specific evidence from district-level facilities. The present study seeks to address this gap by examining delivery outcomes among women with a previous CD at Zithulele Hospital, a rural district hospital primarily staffed by generalist clinicians. Findings from this study will contribute to strengthening clinical governance, informing training priorities and supporting evidence-based decision-making regarding TOLAC in similar rural and district hospital settings.

The study is framed within a maternal safety and systems-capacity model,^14^ recognising that VBAC outcomes are influenced by both individual obstetric risk factors (e.g. parity, cervical dilatation, and/or previous VBAC) and institutional factors (e.g. human, institutional, and/or policy). International guidelines, such as those from the American College of Obstetricians and Gynaecologists^4^ among others, emphasise that VBAC success depends on appropriate patient selection and the ability to safely respond to complications and perform emergency procedures such as abdominal hysterectomy and emergency CD in the management of possible complications that may arise.^4,15^ This framework guided the study design, focusing on measurable delivery outcomes within the realities of a rural district hospital.

This study aims to describe the outcomes and complications – both maternal and neonatal – of labour for women with previous CD managed by generalist doctors at a district hospital (Zithulele Hospital) from 01 January 2021 until sample size was reached. The primary objectives are to describe the final mode of delivery (VBAC or CD – emergent vs. elective) of pregnancies with previous CD, at Zithulele Hospital. Secondary objectives are to describe the maternal and neonatal complications following deliveries in the same cohort.

## Research methods and design

### Study design, population and settings

This study employed a descriptive study design utilising a retrospective record review and analysed Maternity Case Records. This approach enabled structured analysis of routinely collected clinical data from maternity case records without altering patient care.

The study was conducted at Zithulele Hospital, a 147-bed rural district hospital situated in the King Sabata Dalindyebo subdistrict of the OR Tambo District, Eastern Cape, South Africa. The hospital provides 24-h maternity care with on-site emergency surgical capacity and emergency blood transfusion services. Resource constraints include staff shortages, limited access to senior clinicians and emergency transport delays, which reflect the realities of many South African rural, district hospitals. The hospital conducts approximately 2100 deliveries annually, with a CD rate of 22% – 25%.^16^

Zithulele Hospital serves a predominantly rural, low-income population and its obstetric service is carried out by medical officers and/or community-service doctors and midwives, with one family physician overseeing clinical services. Access to hospital remains difficult and expensive for a large proportion of the served population with some reasons being poorly developed road infrastructure and limited household income. This setting provided a unique opportunity to assess delivery outcomes and VBAC feasibility within a rural district health system.

The study population is made up of records of pregnant women arriving at hospital from 01 January to 30 September 2021 and having had a previous CD in a previous pregnancy (Robson score 5).^2^

Inclusion criteria comprised: (1) women with a singleton pregnancy and at least one prior CD; (2) delivery at Zithulele Hospital (vaginally or by CD); and (3) live foetus upon arrival to hospital. Exclusion criteria comprised of: (1) women with absolute contraindications to vaginal delivery in the delivery being assessed (e.g. transverse lie, major placenta praevia, etc.); and (2) incomplete or missing delivery records.

A minimum sample size of 179 records was calculated to provide a representative estimate of outcomes with a 95% confidence level and 5% margin of error. To account for potential exclusions, 20 additional records were reviewed, giving a final dataset of 199 records, of which 195 met inclusion criteria.^17^ All eligible records meeting the criteria during the study period were included.

### Data collection

Data were extracted retrospectively from maternity case records using a standardised data collection form utilising KoboToolbox software (www.kobotoolbox.org). The collection form captured variables across various domains:

- Demographic and Obstetric data: maternal age, parity, antenatal clinic attendance, comorbidities (including HIV, hypertension), number of previous CD and presence of intervening vaginal delivery.
- Current Labour and Delivery: stage of labour at presentation, foetal lie, mode of delivery (VBAC, emergency CD, elective CD), indication for current CD and attending clinician cadre.
- Maternal and Neonatal Outcomes: complications such as postpartum haemorrhage (PPH), sepsis, blood transfusion, uterine rupture, Neonatal Intensive Care Unit (ICU) admission, Apgar scores and perinatal mortality.

Data were captured by the primary investigator into a secure, password-protected spreadsheet and de-identified prior to analysis. Data were exported into Microsoft Excel (2023) for cleaning and analysis. Range and consistency checks were performed to identify outliers, missing values or implausible entries.

Descriptive statistics were calculated for demographic and clinical characteristics. Continuous variables were summarised as means, medians, standard deviations (s.d.) and interquartile ranges (IQR) as appropriate, while categorical variables were expressed as frequencies and percentages.

Comparative analyses were performed to identify factors associated with successful VBAC. Analytical functions were applied including the Chi-square tests for categorical variables (e.g. previous vaginal delivery, number of prior CD, comorbidities). Binary logistic regression (univariate) was used to assess associations between VBAC success and independent predictors, including parity, stage of labour on admission and presence of previous vaginal delivery. A *p*-value < 0.05 was considered statistically significant for all analyses.

As this was a retrospective record review using anonymised data, a waiver of informed consent was granted. All data were handled confidentially, stored securely on password-protected devices and used solely for research purposes.

### Ethical considerations

Ethical approval was obtained from the Walter Sisulu University Faculty of Health Sciences Research Ethics and Biosafety Committee (Reference: 228/2024) and the Eastern Cape Department of Health Research and Ethics Committee. Institutional permission was granted by the Zithulele Hospital Chief Executive Officer.

## Results

A total of 195 maternity records were analysed for the study period (01 January–30 September 2021), exceeding the required minimum sample size of 179. The overall CD rate at Zithulele Hospital during this period was 23.1% (*n* = 426), of which 25% (*n* = 105) of these CD were in women with scarred uteri (Robson score 5).

### Mode of delivery

Among women with previous CD, 46% (*n* = 90) achieved successful VBAC, while 54% (*n* = 105) delivered by CD (Table 1). Of those with successful VBAC, 79% (*n* = 71) were unassisted vaginal deliveries and 21% (*n* = 19) required vacuum assistance. All women with two-or-more-previous-CD (18%, *n* = 35) delivered by repeat CD, either elective or emergency, depending on the onset of labour (considered to be emergency CD if arriving to hospital in established labour).

**TABLE 1:** Baseline characteristics and preliminary findings of women with previous caesarean delivery (*N* = 195).

Characteristic	*n*	%	Mean	s.d	Median	Min	Max	IQR
Age	-	-	27.79	5.11	-	-	-	-
Gravidity	-	-	-	-	3.00	2	7	1
Parity					2.00	2	7	1
**Number of previous caesarean deliveries**								
One	160	82	-	-	-	-	-	-
Two	30	15	-	-	-	-	-	-
Three	5	3	-	-	-	-	-	-
**Previous successful VBAC**								
Not applicable (Gravida 2)	98	50	-	-	-	-	-	-
No	68	35	-	-	-	-	-	-
Yes	29	15	-	-	-	-	-	-
Gestational age (weeks)	-	-	38.95	1.85	-	-	-	-
**Cervical dilation at presentation (cm)**								
0	65	33	-	-	-	-	-	-
1-3	110	56	-	-	-	-	-	-
4-7	13	7	-	-	-	-	-	-
8-10	7	4	-	-	-	-	-	-
**Mode of delivery**								
Elective caesarean	21	11		-	-	-	-	-
Emergency caesarean	84	43		-	-	-	-	-
Spontaneous vaginal	71	36		-	-	-	-	-
Vacuum-assisted vaginal	19	10		-	-	-	-	-
**Intra-operative BTL in currend CD (women with ≥ 2 previous CD)**								
Yes	23	66	-	-	-	-	-	-
No	12	34	-	-	-	-	-	-
**Maternal comorbidities** †								
HIV	91	47	-	-	-	-	-	-
RPR positive	3	2	-	-	-	-	-	-
Hypertension	21	11	-	-	-	-	-	-
Diabetes mellitus	0	0	-	-	-	-	-	-
High BMI	10	5	-	-	-	-	-	-
Other	6	3	-	-	-	-	-	-

† , comorbidities are not mutually exclusive; BTL, bilateral tubal ligation; CD, caesarean delivery; VBAC, vaginal birth after caesarean; s.d., standard deviations; IQR, interquartile ranges; HIV, human immunodeficiency virus; BMI, body mass index; RPR, rapid plasma regain.

Most women in this study with a single-previous-CD (99%, *n* = 158) underwent a TOLAC in hospital, with only two women from this cohort having an elective CD (refusal of TOLAC and maternal intellectual impairment). Overall, 57% (*n* = 90) of patients who underwent TOLAC achieved a successful VBAC.

**FIGURE 1:**
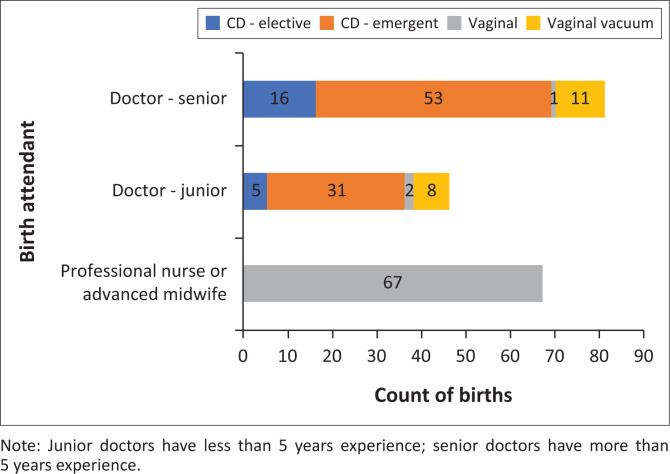
Highest birth attendant cadre attending delivery (*N* = 195).

Of the 90 successful VBACs, the majority (74%, *n* = 67) were attended solely by midwives, while the remaining 26% (*n* = 23) were attended by doctors – most of which were vacuum-assisted deliveries (Figure 1).

### Caesarean deliveries in women with scarred uteri

Among the 105 mothers who delivered by CD in the study population, 20% (*n* = 21) were elective and 80% (*n* = 84) were emergency CD performed after failed TOLAC or for multiple-previous-CD presenting in labour. Elective CD were mainly for multiple prior CD or refusal of TOLAC. Pregnancies with absolute indications (e.g. placenta praevia, footling breech) had been excluded during sampling.

The leading indications for emergency CD are tabulated in Table 2.

**TABLE 2:** Indications for emergency caesarean delivery (*N* = 84).

Indication	*n*	%
Poor progress of labour	30	36.0
Foetal distress	18	21.0
Failed induction of labour	15	18.0
Two or more previous CD presenting in labour	14	16.5
Breech presentation (frank) opting for CD in labour	4	5.0
Inter-CD interval < 18 months/prolonged rupture of membranes/uterine rupture	3	3.5

CD, caesarean delivery.

### Factors associated with vaginal birth after caesarean success

Women with a previous successful VBAC and those presenting in more advanced labour were significantly more likely to achieve VBAC (Table 3). Mean birthweight did not differ significantly between groups.

**TABLE 3:** Comparison of women with caesarean versus vaginal birth after caesarean deliveries.

Variable	CD (*n* = 105)				VBAC (*n* = 90)				*t*	*P*
	Mean	CI	*n*	%	Mean	CI	*n*	%		
Age	28.39	27.39, 29.39	-	-	27.12	26.08, 28.16	-	-	1.74	0.083
Gravidity	2.92	2.70, 3.14	-	-	2.87	2.65, 3.09	-	-	0.33	0.740
Parity	2.83	2.64, 3.03	-	-	2.74	2.53, 2.95	-	-	0.65	0.510
Gestational age	39.03	38.65, 39.41	-	-	38.88	38.52, 39.24	-	-	0.56	0.580
Cervical dilatation (cm)	0.90	0.67, 1.27	-	-	2.47	1.97, 2.96	-	-	−5.26	< 0.001
Birthweight (grams)	3225	3131, 3318	-	-	3114	3026, 3202	-	-	1.70	0.091
Previous successful VBAC (in gravidity of ≥ 3)	-	-	8 (out of 51)	16	21	-	21 (out of 46)	46	*	0.001*

CD, caesarean delivery; VBAC, vaginal birth after caesarean; CI, confidence interval.

### Maternal complications

A total of 26 women (13%) experienced post-delivery complications (Table 4), the majority (69%, *n* = 18) occurring in the CD group. Puerperal sepsis (38%, *n* = 10) was seen exclusively after CD. Adhesions were unique to repeat CD, while perineal tears occurred only in the vacuum-assisted VBAC group. One partial uterine rupture was suspected and confirmed at emergency CD. Only 4% (*n* = 8) required blood transfusion; no maternal deaths occurred.

**TABLE 4:** Maternal complications by delivery mode (*N* = 26).

Maternal complications at & after delivery	CD emergent	CD elective	Vaginal	Vaginal vacuum	Total
Uterine rupture	1	0	0	0	1
PPH/Blood transfusion*	3	1	3	1	8
Puerperal sepsis	8	1	0	0	9
Other (perineal tears, significant adhesions)	5	0	0	3	8

**Total**	**17**	**2**	**3**	**4**	**26**

CD, caesarean delivery.

* , PPH = postpartum haemorrhage; One patient had both PPH requiring blood transfusion and later developed puerperal sepsis.

### Perinatal outcomes

Thirteen neonates (6.7%) required admission to the neonatal unit, most commonly after emergency CD (Table 5). There were no stillbirths or early neonatal deaths recorded during the study period.

**TABLE 5:** Neonatal complications by delivery mode (*N* = 13).

Perinatal complications	CD emergent	CD elective	Vaginal	Vaginal vacuum	Total
Admission to neonatal unit/Low Apgar	0	0	2	1	3
Admission to neonatal unit/Neonatal seizures	3	0	0	0	3
Admission to neonatal unit/other (hypoglycaemia, TTN, etc.)	3	0	3	1	7

**Total**	**6**	**0**	**5**	**2**	**13**

TTN, Transient tachypnoea of newborn, CD, caesarean delivery.

### Additional observations

Because of the increasing risk of uterine rupture in women with higher-order CD, women undergoing their third CD are routinely offered bilateral tubal ligation (BTL) after informing them of the increased risks in any future pregnancy. Of the women counselled for BTL at their third CD, 34% (*n* = 12) declined the procedure despite extensive counselling.

A majority (89%, *n* = 175) presented before or during early labour (≤ 3 cm dilated), which is consistent with health system messaging promoting proactive early arrival to hospital because of long travel distances and limited infrastructure (especially after hours).

Of the total women considered in the study period, those with a previous successful VBAC (15%, *n* = 29) achieved a 72% VBAC success rate, compared with only 37% success among those whose last delivery was by CD.

## Discussion

### Key findings

This study demonstrates that women with scarred uteri (Robson score 5) contribute substantially to the overall CD rate at Zithulele Hospital, accounting for 25% of all CD deliveries during the study period. The VBAC success rate was 46%, which compares favourably with published South African data from tertiary hospitals in Pretoria (36%) and Mpumalanga (42%).^12,13^ However, optimal VBAC success rates internationally are quoted at between 60% and 80%.^18^

Similar patterns have been reported in other rural district hospital settings; for example, a study from KwaZulu-Natal found that 35% of CS occurred in women with previous caesarean sections (Robson group 5), with an improved VBAC success rate of 58% among women undergoing a TOLAC. Together, these findings highlight the significant contribution of women with scarred uteri to overall CS rates while demonstrating that successful VBAC is achievable in rural district hospital settings.^19^

All eligible women (single-previous-CD; no other contra-indications) were offered a TOLAC, and a majority of successful VBACs were attended by midwives. Factors significantly associated with VBAC success were advanced cervical dilatation at presentation (*p* < 0.001) and a previously successful VBAC (*p* = 0.001). Maternal complications occurred in 13% of cases, predominantly in the CD group, while neonatal complications occurred in approximately 7%, with no perinatal deaths.

### Caesarean rate and contribution of scarred uteri

The finding that one quarter of all CD were in women with scarred uteri aligns with global data showing that repeat CD among women with previous scars (Robson score 5) is the single largest contributor to rising CD rates worldwide.^1,2^ Betran et al. and the WHO 2015 report both noted that once a CD is performed, the probability of repeat CD rises sharply, perpetuating a ‘cascade’ of surgical deliveries.^1,2^ This observation has particular relevance for South Africa, where national data show persistently high CD rates in both public and private sectors.^6,20^

The implications are significant for rural district hospitals, where operating theatre capacity and appropriately skilled surgical staff are limited. Repeat CD not only increases the clinical workload, but also exposes women to cumulative risks such as abnormal placentation, haemorrhage and surgical adhesions.^4,21^

### Vaginal birth after caesarean success rate and feasibility in rural settings

The VBAC success rate of 46% in this study compares more favourably to those reported at South African tertiary referral hospitals (36% – 42%), and somewhat below that of an isolated obstetric study done at a rural district hospital in South Africa.^12,13,19^ This may reflect a less complicated patient cohort at district level, as more complex or high-risk pregnancies are referred elsewhere. The finding reinforces that VBAC is both safe and feasible in rural settings when appropriately selected, supervised and supported by clear protocols with access to emergency surgical intervention (including total abdominal hysterectomy) as needed.

This study also confirms the important role of midwives because nearly three-quarters of successful VBACs were attended solely by midwives once decision was made for TOLAC. This illustrates the critical contribution of nurse-led intrapartum care in district-level health facilities in South Africa. Furthermore, the high rate of women agreeable to TOLAC in this study also demonstrates its acceptability and feasibility in a predominantly midwife-managed rural maternity service.

Thus, the study findings align with the RCOG 2015 among other guidelines, which recommend offering VBAC to women with a single previous lower-segment CD and no contraindications.^22^

### Indications for repeat caesarean delivery

A large proportion (79%) of CD in this study were indicated for reasons directly related to the uterine scar – including multiple-previous-CD and short inter-pregnancy interval – both of which are well-recognised risk factors for uterine rupture.^4^ Furthermore, restricted options for safe induction or augmentation in women with scarred uteri meant that poor labour progress often resulted in repeat CD, again illustrating the *Caesars-beget-Caesars* phenomenon.^4^ These findings are consistent with international evidence and emphasise how structural and clinical limitations within rural hospitals may further perpetuate repeat surgical deliveries.^6^

### Factors influencing vaginal birth after caesarean success

The strong association between advanced cervical dilatation at presentation and successful VBAC is consistent with existing evidence.^12,13^ Women arriving in established labour are more likely to achieve vaginal delivery, whereas those admitted in latent or early labour are more susceptible to interventions possibly leading to CD.

Similarly, a previous successful VBAC was a robust predictor of VBAC success (*p* = 0.001). This supports international evidence showing that a prior vaginal birth, especially a previous VBAC, significantly increases the likelihood of subsequent VBAC.^12,22^ These findings provide valuable guidance for risk stratification and counselling at the district level.

In contrast, birthweight and parity were not significantly associated with VBAC success in this cohort, differing from other studies that link lower birthweights and higher parity to increased VBAC rates.^12^ The relative homogeneity of the study population and smaller sample size may explain this difference.

### Maternal and neonatal complications

The overall maternal complication rate (13%) was low and predominantly associated with CD, echoing national maternal mortality reports showing higher morbidity in surgical deliveries.^6,20^ Puerperal sepsis was the most frequent complication, confined to the CD group, underscoring the unique additional risks post CD delivery. Adhesions encountered during repeat CD procedures highlight the cumulative risk of multiple surgeries, particularly where junior, inexperienced doctors perform most operations.

The incidence of uterine rupture (0.5%) was low and comparable to published global rates for optimally managed TOLAC (0.5% – 0.7%).^4,22^ Importantly, there were no maternal or neonatal deaths, supporting the safety of VBAC when conducted according to published guidelines and with access to appropriately skilled clinicians. Neonatal complications were mild and evenly distributed, with no difference in Apgar outcomes between VBAC and CD groups.

### Cultural considerations and family planning

A notable observation was that one-third of women eligible for BTL declined the procedure despite counselling on the risks of higher-order CD. This suggests that preserving fertility and having larger families remains a very strong cultural priority in this rural setting. However, this has important public health implications, as each successive CD increases the risk of placenta accreta and massive haemorrhage as well as uterine rupture in further pregnancies.^4,21,23^

### Strengths and limitations

A key strength of this study is that it provides real-world evidence from a rural district hospital, a setting underrepresented in South African obstetric literature. The dataset captures all women with previous CD who completed delivery at Zithulele over a defined period, ensuring comprehensive inclusion and minimising sampling bias.

However, as a retrospective record review, the study relied on the accuracy and completeness of clinical documentation. Potential underreporting of minor complications and missing data on certain variables (e.g. duration of labour, exact indication for prior CD) may have limited further subgroup analysis and may be a potential source of bias in the study. Additionally, the sample size, though adequate for descriptive analysis, restricted the ability to perform multivariate modelling of predictors.

Despite these limitations, the findings remain generalisable to other rural hospitals in South Africa with similar service structures and patient demographics.

### Implications for practice and policy

This study provides actionable insights for district-level obstetric care:

- **Support and awareness for TOLAC programmes:** With nearly half of women achieving successful VBAC and minimal complications, TOLAC should remain an encouraged and monitored practice in rural settings.
- **Midwife empowerment and training:** Given the high proportion of VBACs attended by midwives, continued training in intrapartum monitoring and early complication recognition is critical.
- **Doctor experience and training:** The high number of complicated CD done in district level (in women with scarred uteri) as well as the inherent – albeit uncommon – risks of appropriately selected VBAC, calls for upskilling of junior doctors and ensuring availability of senior doctors – such as family physician – in these centres. This ensures the ability to adequately manage any arising complications or complex surgeries.
- **Further research:** Multicentre studies across district hospitals could further assess the impact of generalist-led obstetric care on outcomes.

## Conclusion

Women with previous CD (Robson score 5) contribute significantly to overall CD rates at Zithulele Hospital. Nearly half of these women achieved successful VBAC, a result comparable to tertiary-level outcomes. The study demonstrates that VBAC is a safe and feasible delivery option in rural district hospitals, provided that women are appropriately selected and supported by trained staff with access to emergency surgical care.

Maternal complications were low and predominantly associated with CD, reinforcing the importance of avoiding unnecessary repeat operations. The identified predictors – previous successful VBAC and advanced labour on admission – offer practical guidance for risk assessment and patient counselling in rural obstetric practice.

Promoting VBAC where clinically appropriate can contribute to reducing the escalating CD rate, conserving surgical resources and improving maternal outcomes in South Africa’s district health system. Further research in similar contexts is warranted to expand the evidence base and inform national policy.
